# Enhancing Radiation Dose Efficiency in Prospective ECG-Triggered Coronary CT Angiography Using Calcium-Scoring CT

**DOI:** 10.3390/diagnostics13122062

**Published:** 2023-06-14

**Authors:** Muhammad Taha Hagar, Martin Soschynski, Matthias Benndorf, Thomas Stein, Jana Taron, Christopher L. Schlett, Fabian Bamberg, Tobias Krauss

**Affiliations:** 1 Department of Diagnostic and Interventional Radiology, Medical Center—University of Freiburg, Faculty of Medicine, University of Freiburg, 79085 Freiburg, Germany; martin.soschynski@uniklinik-freiburg.de (M.S.); matthias.benndorf@klinikum-lippe.de (M.B.); thomas.stein@uniklinik-freiburg.de (T.S.); jana.taron@uniklinik-freiburg.de (J.T.); christopher.schlett@uniklinik-freiburg.de (C.L.S.); fabian.bamberg@uniklinik-freiburg.de (F.B.); tobias.krauss@uniklinik-freiburg.de (T.K.); 2 Department of Diagnostic and Interventional Radiology, Medical Faculty OWL, University of Bielefeld, Klinikum Lippe, 32756 Detmold, Germany

**Keywords:** coronary CT angiography, calcium scoring, radiation dose

## Abstract

Background: This study investigates whether the scan length adjustment of prospectively ECG-triggered coronary CT angiography (CCTA) using calcium-scoring CT (CAS-CT) images can reduce overall radiation doses. Methods: A retrospective analysis was conducted on 182 patients who underwent CAS-CT and prospectively ECG-triggered CCTA using a second-generation Dual-Source CT scanner. CCTA planning was based on CAS-CT images, for which simulated scout view planning was performed for comparison. Effective doses were compared between two scenarios: Scenario 1—CAS-CT-derived CCTA + CAS-CT and Scenario 2—scout-view-derived CCTA without CAS-CT. Dose differences were further analyzed with respect to scan mode and body mass index. Results: Planning CCTA using CAS-CT led to a shorter scan length than planning via scout view (114.3 ± 9.7 mm vs. 133.7 ± 13.2 mm, *p* < 0.001). The whole-examination effective dose was slightly lower for Scenario 1 (3.2 [1.8–5.3] mSv vs. 3.4 [1.5–5.9] mSv; *p* < 0.001, *n* = 182). Notably, Scenario 1 resulted in a significantly lower radiation dose for sequential scans and obese patients. Only high-pitch spiral CCTA showed dose reduction in Scenario 2. Conclusions: Using CAS-CT for planning prospectively ECG-triggered CCTA reduced the overall radiation dose administered compared to scout view planning without CAS-CT, except for high-pitch spiral CCTA, where a slightly opposite effect was observed.

## 1. Introduction

Cardiovascular disease is the leading cause of mortality among adults worldwide [1]. Early diagnosis, risk adjustment, and treatment have improved patient outcomes [2]. Coronary CT angiography (CCTA) represents a reliable and accurate non-invasive imaging technique for detecting coronary artery disease (CAD) with a sensitivity of 96% and a specificity of 82% [3,4,5,6]. In the updated guidelines of the European Society of Cardiology, CCTA received a class-I recommendation for patients with a low to intermediate pretest probability for CAD [7]. 

Despite recent advancements in CCTA, concerns remain regarding radiation exposure. The Society of Cardiovascular CT has published guidelines for optimized CCTA acquisitions to minimize radiation exposure while maintaining diagnostic accuracy [8]. Some such strategies include tube voltage reduction for underweight and normal-weight patients [9,10], iterative reconstruction techniques [11], z-axis adaption [12], artificial-intelligence-based patient positioning [13], and deep-learning-based scan range planning [14]. Lastly, a proposed approach for reducing radiation dose is to omit non-contrast calcium-scoring CT (CAS-CT) before contrast-enhanced CCTA [15]. 

With the emergence of second-generation Dual Source CT technology (DSCT), two novel, low-dose, prospectively ECG-triggered scan modes have been made available: sequential (“step and shoot”) and high-pitch spiral (“flash”) CCTA [16]. As retrospective ECG-gated CCTA is now being increasingly supplanted by these modern, prospective, ECG-triggered DSCT protocols [17], the impact of calcium-scoring CT omission on whole-examination radiation doses remains unexplored. 

Thus, our study aims to examine the influence of the scan length adjustment of low-dose, prospectively ECG-triggered CCTA using CAS-CT and determine whether the omission of CAS-CT represents a viable strategy for reducing the radiation dose of the whole examination.

## 2. Materials and Methods

### 2.1. Ethics Statement

Approval was obtained on 11 March 2021 (No. 21-1060) by the institutional review board of the University of Freiburg Medical Center, and the need for informed consent was waived. 

### 2.2. Patients

Between January 2020 and September 2020, consecutive patients clinically referred for CCTA were retrospectively included in the study group. The following data on patient characteristics were recorded: gender, age, height, weight, clinical symptoms, pretest probability for CAD, blood pressure, and heart rate. Exclusion criteria included a deviating or incomplete examination protocol, scan abortion, and an already-known CAD. 

### 2.3. CT Protocols 

All CT examinations were conducted using a 2nd-generation DSCT scanner (Somatom Definition Flash, Siemens Healthineers, Forchheim, Germany). Initially, an anterior–posterior scout view of the chest was obtained. The craniocaudal extension of CAS-CT was then planned using the scout view, with an upper limit of 1 cm below the carina and a lower limit of the apex cordis. Non-contrast CAS-CT was performed using a low-dose, high-pitch scan mode with the following parameters: tube voltage 120 kV, automated tube current modulation (CARE Dose mAs at a reference of 80 mAs), detector collimation of 2 × 128 × 0.6 mm, gantry rotation time of 280 ms, a pitch of 3.2, and a matrix size of 512 × 512. Images were acquired at 60% of the R-R interval during an inspiratory breath-hold, for which patients were trained by a doctor before each scan. Images were transferred with a default mediastinal window setting, a slice thickness of 3.0 mm, and a field of view restricted to the heart, and a sharp reconstruction kernel was applied (b36f). 

After CAS-CT, a beta-blocker (metoprolol, 1 mg/mL) was administered intravenously if necessary, and patients received a single dose of 5.0 mg of isosorbide-dinitrate if there were no contraindications. The planning of the craniocaudal extension for CCTA was carried out using transversal CAS-CT images. To achieve this, one radiologist and one expert technician identified the table position of the highest point of the coronary tree, primarily the proximal left anterior descending artery, as well as the apex cordis by consensus. An additional 1 cm was added to these landmarks for the extension in order to account for variability in patient breathing. 

A test-bolus preceded CCTA to determine the time delay until maximal aortic contrast enhancement; accordingly, a region of interest was placed in the aortic root, and consecutive image acquisition started 10 s after intravenous injection of 10 mL of iodinated contrast material (Iomeprol, 400 mg/dL) followed by 60 mL using a dual-syringe power injector. The time to attenuation peak was measured, and 4 s were added to the time until peak in sequential scan mode and 5 s in high-pitch spiral scan mode to determine the CCTA scan delay [18]. 

For CCTA, 60-72 mL of contrast material (Imeron 400, Bracco imaging, 400 mg iodine/mL, injection rate: 6 mL/s) was injected, followed by a 60 mL saline flush (injection rate: 6 mL/s). A patient-specific simple score, calculated based on our institutional standards of procedure for CCTA acquisition [18], was used to determine the specific prospective ECG-triggered scan protocol (either high-pitch spiral CCTA or sequential CCTA). This acquisition score considers the patient’s heart rate, heart rate variability, body composition, and pretest probability of obstructive CAD [7,19]. Detailed metrics of the score-based CCTA acquisition recommendations are provided in Table A1 and Table A2. 

When a sequential scan was applied, images were reconstructed in the automatically determined best diastolic or, if applicable, best systolic phase and in a multiphase dataset with 5% steps R-R interval [18]. All CCTA images were reconstructed using a vascular convolution kernel (Bv48), a slice thickness of 0.6 mm, and an increment of 0.4 mm. A field of view restricted to the heart of 180 × 180 mm was applied. Expert technicians, supervised by a doctor, acquired all CT images using breath-holding techniques.

### 2.4. CT Data Analysis 

Two radiologists with 4 (M.T.H.) and 9 years (M.S.) of experience in cardiovascular CT analyzed all images by consensus on a dedicated workstation (Syngo.via, software Version VA50, Siemens Healthineers). They were blinded to clinical history and prior imaging examinations. Any disagreements were resolved by involving a third radiologist with 15 years of experience in cardiovascular CT (T.K.). 

#### 2.4.1. Evaluation of CCTA Image Quality

**Figure A1:**
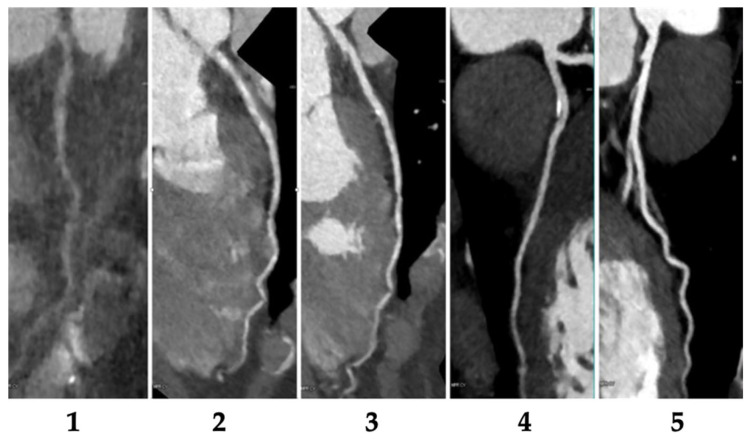
Curved multiplanar reformations of the left anterior descending artery serve as an example of the utilized 5-point Likert scale to evaluate subjective image quality. The Likert scale ranges from 1, indicating “nondiagnostic” image quality, to 5, representing excellent image quality, for the assessment of coronary vessels.

Image quality was assessed with respect to the coronary tree’s vessel sharpness, movement artifacts, and contrast attenuation. A five-point Likert scale was applied for image quality assessment, whose scoring details are as follows: 1 = “non-diagnostic” (extensive artifacts and vessel deformation), 2 = “fair” (many artifacts present yet determined diagnostic by consensus), 3 = “moderate” (blurred vessel contours and numerous artifacts), 4 = “good” (slight radiating artifacts), and 5 = “excellent” (crisp and smooth vessel wall contours and no artifacts). An example is provided in Figure A1.

#### 2.4.2. Determination of Scan Range 

The craniocaudal extension of CAS-CT was then planned from the anteroposterior scout view, with an upper limit of 1 cm below the carina and a lower limit of the apex cordis. The scan length of CAS-CT and the scan length of CCTA were determined by consensus involving one radiologist and one expert technician. In this process, the table position of the first image above the most cranial part of the coronary tree and the first image below the cardiac apex were noted. CCTA was planned by adding 1 cm to the upper and 1 cm to the lower cardiac border on CAS. The scan length of the simulated scout-view-based CCTA was assumed to be equal to the CAS-CT. Thus, the table positions of the uppermost and lowest axial images of calcium scoring were noted and used to calculate the scout-view-derived scan length of CCTA, as previously described [20]. 

#### 2.4.3. Assessment of Radiation Dose 

The dose-length product (DLP) and volume CT dose index (CTDIvol) were noted fromthe scan protocol recorded in each CT examination. According to the method defined by the European Working Group for Guidelines on Quality Criteria for Computed Tomography, the effective dose was calculated by multiplying the DLP by an averaged conversion coefficient (k = 0.017 mSv × mGy^−1^ × cm^−1^) between both sexes through Monte Carlo simulations [21]. The radiation dose estimates for CAS-CT and CCTA, with a scout-view-derived scan length and a CAS-CT-derived scan length, were calculated. 

### 2.5. Statistical Analysis 

IBM SPSS Statistics for Macintosh (Version 28.0, Armonk, NY, USA) and MedCalc Statistical Software (Version 15.8, Ostend, Belgium) were used for statistical analysis. First, we used graphical Q–Q plots and the Shapiro–Wilk test to determine whether the variables followed a normal distribution. Continuous variables are expressed as numbers, frequencies are given in parenthesis, and mean ± standard deviation (SD) or median and interquartile range are presented in square brackets, as appropriate. CCTA scan length derived from CAS-CT and simulated CCTA scan length derived from anteroposterior scout view were compared using paired t-test. The following radiation dose scenarios were calculated and compared using the Wilcoxon signed-rank test: Scenario 1 = CAS-CT-derived CCTA + CAS-CT and Scenario 2 = scout-view-derived CCTA without CAS-CT. Furthermore, to assess the association between radiation dose and clinical and technical scan parameters, we fitted linear regression models, including patients’ gender, body mass index (BMI), tube voltage, and scan range. Radiation dose served as the outcome of interest, while clinical and technical parameters served as covariates. Radiation dose differences between Scenarios 1 and Scenario 2 were further compared in stratified subgroups with respect to applied prospective ECG-triggered scan mode–high-pitch spiral CCTA and sequential CCTA, applied tube voltage, and body mass index. A two-tailed *p*-value of <0.05 was considered statistically significant for all tests. 

## 3. Results

### 3.1. Patients’ Characteristics

**Figure 1:**
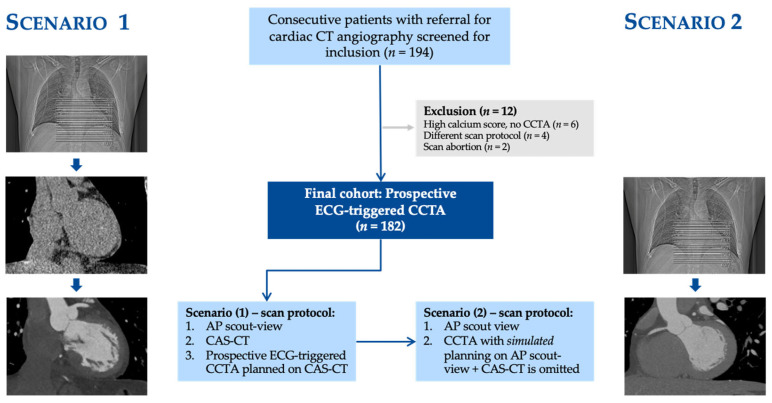
Flowchart of patient inclusion and exclusion criteria: in Scenario 1, every patient was imaged using an anteroposterior scout view, calcium-scoring CT that was planned based on the scout view, and CCTA with a scan range planned using calcium-scoring CT. In Scenario 2, we simulated a CCTA scan for every patient, with the scan range planned based on the anteroposterior scout view, while omitting calcium-scoring CT. Abbreviations: ECG, electrocardiogram; CCTA, coronary computed tomography angiography; CAS-CT, coronary artery calcium-scoring computed tomography.

A total of 182 patients (mean age 58 ± 12 (SD) years; 47% females) were included. High-pitch spiral CCTA was performed for 18% (32/182) of the subjects and sequential CCTA was performed for 82% (150/182). Patients’ characteristics are summarized in Table 1, while a flowchart concerning patient inclusion and exclusion criteria and the study’s design is provided in Figure 1. 

### 3.2. Evaluation of Image Quality 

The overall diagnostic image quality was good (with a median Likert score of 4 [IQR, 3–5]). Excellent image quality was achieved frequently (66/182 (36%)). In 5 out of 182 examinations (3%), diagnostic image quality could not be achieved (Likert score 5). In four of the five patients (80%), the midpart of the right coronary artery was not assessable due to severe motion artifacts. In every one of these patients, a high-pitch spiral CCTA scan was employed. 

### 3.3. Determination of Scan Range 

**Figure 2:**
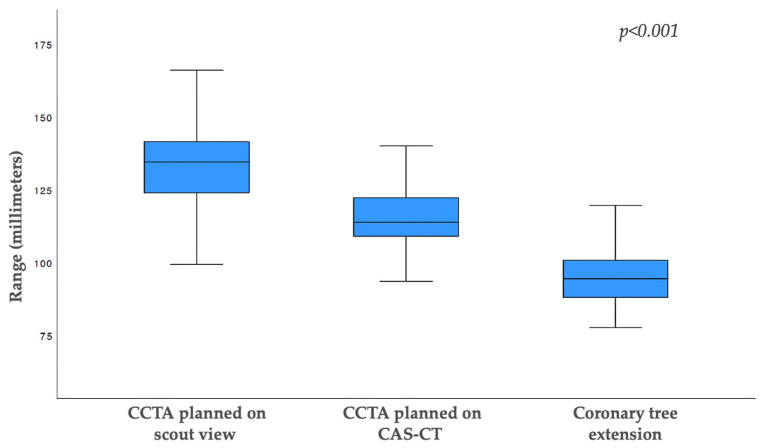
Boxplot showing the scan length distribution of scout view-based coronary CT angiography (133 ± 13.2 mm), calcium-scoring CT-based coronary CT angiography (114.6 ± 9.8 mm), and the craniocaudal coronary tree extension on the acquired CT images (94.7 ± 8.7 mm). Length differences for each entity were significant (*p* < 0.001). The entire coronary tree was depicted in all patients for each planning approach. Abbreviations: CCTA, coronary computed tomography angiography; CAS-CT, coronary artery calcium-scoring computed tomography.

In 166 out of 182 (91%) CT examinations, CAS-CT-derived CCTA scans were found to have a shorter craniocaudal scan length than CCTA planned using anteroposterior scout view images. On average, planning CCTA using scout view images resulted in an 18% longer scan range than planning CCTA using CAS-CT (133.7 ± 17.8 mm vs. 114 + 9.7 mm; *p* < 0.001). However, for both planning methods, the coronary tree, which showed a craniocaudal extension of 94.7 ± 8.7 mm, was fully displayed (Figure 2). 

### 3.4. Assessment of Radiation Dose 

**Figure 3:**
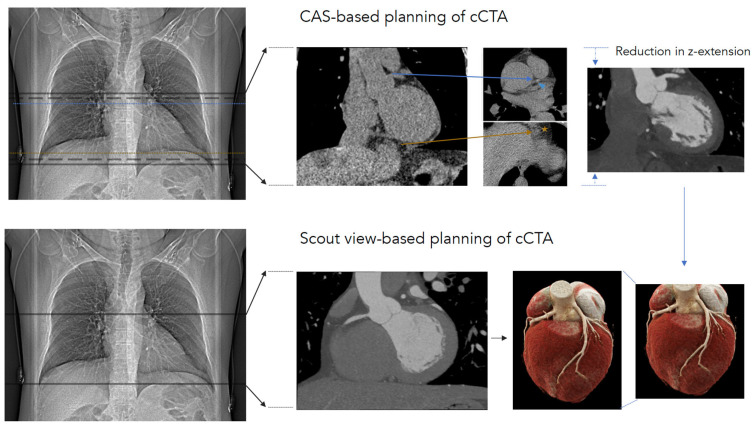
To obtain a complete depiction of the coronary tree, either calcium-scoring CT-based or scout-view-based planning is feasible. With regard to calcium-scoring CT-based planning (upper row), a calcium-scoring CT scan was planned in anterioposterior (AP) scout view presenting an image 1 cm below the carina and 1 cm below the cardiac apex (solid lines on AP scout view). In the calcium-scoring images, the most cranial part of the coronary tree (blue arrowhead) and the cardiac apex (orange star) are depicted (dotted lines in AP-scout view). CT coronary angiography scan length was set at 1 cm with respect to the cranial and caudal regions of those landmarks (dashed lines on AP-scout view). In scout view-based coronary CT angiography planning (lower row), the scan length was planned based on the scout view, with 1 cm below the carina and 1 cm below the cardiac apex defined as landmarks. The calcium-scoring CT scan itself can be omitted in such an approach.

CCTA planning achieved using scout view images showed a 13% higher DLP compared to planning via axial CAS-CT images (198 [94–345] mGy*cm vs. 174 [84–293] mGy*cm; *p* < 0.001). The radiation dose of CAS-CT itself was 20 [16,17,18,19,20,21,22] mGy*cm. In Scenario 1, the acquisition of CAS-CT is mandatory, while in Scenario 2 (CCTA planned using scout-view images), CAS-CT can be omitted (Figure 3). The results of the multiple linear regression model, which was created to identify subgroups for further stratification, are provided in Table A3.

The total DLP comparison between Scenario 1 (CAS-CT derived CCTA + CAS-CT) and Scenario 2 (scout-view-derived CCTA without CAS-CT) resulted in a 3.6% lower DLP for Scenario 1: 191 [103–314] mGy*cm vs. 198 [94–345] mGy*cm (*n* = 182, *p* < 0.001).

When analyzing the subgroups stratified by specific prospective ECG-triggered CCTA scan protocols, it was observed that the differences between Scenario 1 and Scenario 2 were more pronounced. In sequential CCTA (*n* = 150), Scenario 1 achieved a radiation dose reduction of 5.9%, i.e., 214 [138–359] mGy*cm vs. 227 [141–385] mGy*cm (*p* < 0.001). With ECG-padding of 20% or more of the R–R interval applied to sequential CCTA (*n* = 99), Scenario 1 resulted in a radiation dose reduction of 11%, i.e., 241 [164–431] mGy*cm vs. 269 [188–464] mGy*cm (*p* < 0.001). In contrast, for high-pitch spiral CCTA (*n* = 32), Scenario 2 demonstrated a lower radiation dose than Scenario 1, namely, 48 [40–80] mGy*cm vs. 57 [48–88] (*p* < 0.001). The results are summarized in Table 2. Scenario 1 presented a relevant radiation dose reduction in obese patients (*n* = 45) (398 [264–530] mGy*cm vs. 427 [274–584] mGy*cm; *p* < 0.001) or when a tube voltage of 120 kV was applied, namely, 359 [229–490] mGy*cm vs. 373 [222–527] mGy*cm (*p* < 0.001).

## 4. Discussion 

In our study, we examined whether the inclusion of CAS-CT into a low-dose, prospectively ECG-triggered CCTA protocol would result in a whole-examination radiation dose reduction compared to an alternative approach where prospectively ECG-triggered CCTA is planned using scout view images and no CAS-CT is acquired. The essential findings of our study can be summarized as follows: (I) consistently planning the scan range of CCTA using axial non-contrast CAS-CT images results in a shorter scan length than planning using a scout view. (II) The whole-examination radiation dose of prospective ECG-triggered CCTA planned via CAS-CT is slightly lower than CCTA planned using scout view, even if no CAS-CT is acquired in the latter scenario. (III) The CAS-CT planning of prospective ECG-triggered CCTA results in a radiation dose salvage compared to scout view planning when individual factors (e.g., high BMI) or technical factors (e.g., high tube voltage or sequential scan with extended ECG-padding) contributing to a higher overall radiation dose are present. (IV) Omitting CAS-CT to lower the radiation dose of cCTA is not a reasonable strategy because the potential to reduce the CCTA scan range and, consequently, the radiation dose cannot be exploited. (V) In this regard, the acquisition of only low-dose high-pitch spiral CCTA constitutes an exception. 

### 4.1. Scan Range

Our results show that planning CCTA using CAS-CT images results in a shorter scan length than planning using scout view images (133.7 ± 17.8 mm vs. 114.3 ± 9.7 mm; *p* < 0.001). These results confirm those of a previous study using the same CCTA-planning approach (their reported result: 139 ± 13 mm for cCTA using scout view planning vs. 117 ± 9 mm for CCTA using CAS-CT planning [20]). 

### 4.2. Radiation Dose

In our study, CCTA images were acquired using modern and low-dose prospective ECG-triggered scan protocols; consequently, we observed a median overall radiation dose of 3.2 [1.8–5.3] mSv. A study conducted by Leschka et al. on the same scanner observed an overall radiation dose of 9.0 ± 0.6 mSv for CCTA [20]. The differences can be explained by the fact that they applied retrospective ECG-gated CCTA scanning protocols, which are associated with elevated radiation exposure [22]. In their study, the overall radiation dose reduction of CCTA, when planned using CAS-CT instead of scout view images, was 16% [20], whereas we only observed a 4% reduction. These differences can be explained by the fact that the adjustment of the scan range seemed especially effective when the overall radiation dose was elevated. In our study, the median overall dose length product of 174 mGy*cm for CCTA was comparable to that reported in the current registry data (their reported median dose: 195 mGy*cm) [17]. 

### 4.3. Calcium-Scoring CT

Our study’s effective dose for the calcium-scoring scan was 0.33 mSv (IQR, 0.25–0.37 mSv), which is slightly lower than in previous comparable studies [23]. One explanation for this result could be that we always acquired our CAS-CT images using a high-pitch spiral scan, while in other studies, a sequential scan was obtained if the heart rate exceeded 80 bpm. It should be noted that with the emergence of third-generation DSCT and tin filtration, the acquisition of high-pitch, low-voltage CAS-CT images is feasible and results in much lower radiation exposure (0.13 mSv) than in our study [24]. In addition to being a planning tool, non-contrast CAS-CT itself adds to the benefits of additional diagnostic and prognostic value. In a previous study incorporating 13,644 individuals, it was demonstrated that the use of statins in patients suffering from hyperlipoproteinemia only led to a reduction in serious adverse cardiovascular events if coronary calcification was present, indicating its important role as a prognostic marker guiding therapy [25]. It has also been observed that knowledge of calcium scores increases patients’ compliance with statin medication [26]. Therefore, on case-by-case basis, it must be critically considered whether the omission of CAS-CT seems justifiable for radiation safety reasons. 

### 4.4. Limitations

Our study has several limitations. First, this was a retrospective, single-center, single-scanner trial. Therefore, the generalizability of the results is, by definition, limited. Second, all scout view CCTA plans were simulated, and a simulated scan length and radiation dose were calculated. In this respect, a prospective study design would have been more accurate and provided further value. Third, all the participants were included consecutively, and CCTA was clinically indicated; however, selection bias cannot be entirely excluded. Fourth, the analyzed prospective ECG-triggered scan protocols are vendor-specific. Lastly, due to the non-invasive nature of our study, we did not acquire invasive coronary angiography data and, therefore, were unable to assess the diagnostic accuracy of the proposed protocols against an invasive reference standard.

However, our study is the first to investigate the role of CAS-CT as a planning tool for optimizing whole-examination radiation doses in low-dose modern prospective ECG-triggered CCTA. Prospective ECG-triggered CCTA acquisitions have replaced retrospective spiral scans in most clinical settings [17]. 

## 5. Conclusions

Planning prospectively ECG-triggered CCTA via CAS-CT can potentially reduce the overall radiation dose of an examination compared to a scout view planning approach wherein no CAS-CT is acquired. A slightly opposite effect was only observed for high-pitch spiral CCTA. 

## Figures and Tables

**Table 1:** Patients’ characteristics.

Characteristics	Values
Number of subjects	182 (100%)
Age (years)	58 ± 12
**Gender**	
Male	97 (53.3%)
Female	85 (46.7%)
Body mass index (kg/m^2^)	
Non-obese (BMI < 30)	132 (74.9%)
Obese (BMI ≥ 30)	45 (25.1%)
Heart rate during CCTA (bpm)	62 ± 10
**Symptoms**	
Angina pectoris	18 (9.9%)
Atypical angina pectoris	19 (10.4%)
Non-anginal chest pain	47 (25.8%)
Dyspnea	21 (11.5%)
Other	77 (42.3%)
Tube voltage (kV)	
80 kV	22 (12.1%)
100 kV	89 (48.9%)
120 kV	71 (39.0%)
**Prospective ECG-triggered CCTA scan mode**	
High-pitch spiral CCTA	32 (17.6%)
Sequential CCTA	150 (82.4%)
ECG-padding of sequential CCTA	
ECG-window ≤ 20% of the R–R interval	51/150 (33.3%)
ECG-window > 20% of the R–R interval	99/150 (66.7%)
Agatston-Score *	0.2 [0–36]
**CCTA results**	
No CAD	130 (71.4%)
1-vessel CAD	40 (22%)
2-vessel CAD	9 (4.9%)
3-vessel CAD	3 (1.6%)

Data are presented as numbers with frequencies in parentheses or proportions and frequencies in parentheses along with mean ± standard deviation. Abbreviations: BMI, body mass index; CCTA, coronary CT angiography; CAD, coronary artery disease; ECG, electrocardiogram; kV, kilovolt; bpm, beats per minute; R–R interval, elapsed time percentage between two R waves of the QRS signal on the electrocardiogram. * refers to values presented in median and interquartile ranges in square brackets.

**Table 2:** Assessment of Radiation Dose.

	Scenario 1	Scenario 2	*p*-Value
Total (*n* = 182)			
CTDIvol (mGy)	15.3 [7.3–28.5]	15.3 [7.3–28.5]	NS
DLP (mGy*cm)	191 [103–314]	198 [94–345]	*p* < 0.001
Effective dose (mSv)	3.2 [1.8–5.3]	3.4 [1.5–5.9]	*p* < 0.001
Sequential scan mode (*n* = 150)			
CTDIvol (mGy)	18.4 [10.9–31]	18.4 [10.9–31]	NS
DLP (mGy*cm)	214 [138–359]	227 [141–385]	*p* < 0.001
Effective dose (mSv)	3.6 [2.3–6.1]	3.9 [2.4–6.5]	*p* < 0.001
High-pitch spiral scan mode (*n* = 32)			
CTDIvol (mGy)	3.3 [3.0–5.8]	3.3 [3.0–5.8]	NS
DLP (mGy*cm)	57 [48–88]	48 [40–80]	*p* < 0.001
Effective dose (mSv)	1.0 [0.8–1.5]	0.8 [0.6–1.4]	*p* < 0.001

Data are presented as median and interquartile ranges given in square brackets, as appropriate. Abbreviations: CAS-CT, calcium-scoring computed tomography; CCTA, coronary computed tomography angiography; DLP, dose length product; CTDIvol, computed tomography dose index; NS, non-significant. Notes: Scenario 1 incorporates the radiation dose of calcium-scoring CT (DLP 20 [16–22] mGy*cm) and calcium-scoring CT-derived CCTA, whereas Scenario 2 incorporates only the radiation dose of scout-view-derived CCTA.

## Data Availability

The data presented in this study are available on request from the corresponding author.

## Appendix A

These supplementary tables provide a standardized scoring system and recommendations for conducting CCTA on patients with varying cardiovascular risk factors.

Table A1 outlines a simple score based on five criteria: heart rate, elevated heart rate variability, body composition, coronary plaque [27], and pretest probability of obstructive coronary artery disease (CAD) [19]. Each criterion is assigned a point value depending on the patient’s specific condition, which, when summed, determines the most appropriate scanning mode for CCTA. Table A2 details the scan mode recommendations according to the sum score, ranging from high-pitch spiral CCTA to retrospective spiral CCTA or an alternative diagnostic approach. This scoring system and its subsequent recommendations aim to optimize the CCTA process for patients, ensuring the most accurate and efficient assessment of their cardiovascular condition and tailoring patient-specific acquisition, as recommended in the recent guidelines of the Society of Cardiovascular CT [8] and our institutional experience [18].

**Table A1:** Standardized coronary CT angiography using a simple score.

**Criteria**	**1-Point**	**2-Points**	**3-Points**
Heart Rate (bpm)	<65	65–80	>80
Elevated heart rate variability *	No	Yes	Absolute Arrhythmia
Body mass index (kg/m^2^)	≤25	25–30	≥30
Coronary plaque ^†^	None/Minimal (CACS 0–100)	Moderate (CACS 101–300)	Severe (CACS > 301)
Pretest probability of obstructive CAD ^¥^ (%)	0–15	>15	

Abbreviations: bpm, beats per minute; CACS, coronary artery calcium score (Agatston Score). * Defined as >6 beats per minute. ^†^ Using the same visual or quantitative criteria as proposed in recent CAD-RADSTM 2.0 guidelines [27]. ^¥^ Derived from a pooled analysis of 15,815 individuals and according to patient’s gender, age, and symptoms [19].

**Table A2:** Score-derived CCTA prospective ECG-triggered scan protocol recommendations.

**Sum Score**	**Scanning Recommendation**
4–6 points	High-pitch spiral CCTA
7–8 points	Sequential CCTA
9–12 points	Sequential CCTA with extended ECG–padding
13–14 points	Retrospective Spiral CCTA or alternative diagnostic approach

## Appendix C

**Table A3:** Multiple linear regression model with radiation dose acting as the dependable variable.

	**Radiation Dose of CCTA**		
**Linear Regression**	**β**	***p*-Value**	**95% CI**
Gender	0.08	0.25	(−40.6, 70.7)
Age	0.20	0.005	(1.0, 5.6)
BMI	0.39	0.001	(6.8, 19.3)
Tube Voltage	0.47	<0.001	(3.8, 9.0)
Scan Range	0.18	0.014	(0.8, 6.8)

The overall *p*-value of the model was significant (*p* = 0.01). Abbreviations: CI, Confidence interval; BMI, body mass index. Data in parentheses represent the 95% confidence interval range.
